# Applications, benefits and challenges of telehealth in India during COVID-19 pandemic and beyond: a systematic review

**DOI:** 10.1186/s12913-022-08970-8

**Published:** 2023-01-04

**Authors:** Eslavath Rajkumar, Aswathy Gopi, Aditi Joshi, Aleena Elizabeth Thomas, N. M. Arunima, Gosetty Sri Ramya, Prachi Kulkarni, P. Rahul, Allen Joshua George, John Romate, John Abraham

**Affiliations:** 1 Department of Psychology, Central University of Karnataka, Kalaburagi, Karnataka India; 2 Humanities and Applied Sciences, Indian Institute of Management, Ranchi, Jharkhand India; 3 St. John’s Medical College, Bangalore, Karnataka India

**Keywords:** Telehealth, Mobile health, Remote consultation, COVID-19, India, Systematic review

## Abstract

**Background:**

India, the seventh-largest country in the world and the second-most populated faces enormous challenges when it comes to healthcare. The country’s healthcare system was close to collapse due to the detrimental effects of the COVID-19 pandemic. Telehealth, which enables treating patients remotely, played a critical role during these challenging times. This systematic review investigates in detail the role of telehealth during COVID-19 and its application beyond the pandemic.

**Methods:**

Database searches on PubMed, Scopus, Science Direct and Web of Science were carried out for studies published on telehealth, and articles were included if they focused on any audio or video telehealth consultation during the pandemic in India. Findings were synthesised into three main themes: applications, benefits and challenges of telehealth services. Methodological quality was assessed using JBI critical appraisal tools.

**Results:**

The initial search on databases yielded 1143 articles. Of those, 19 met the eligibility criteria. Findings highlight the effective utilisation of telehealth across multiple medical specialities. Although insufficient technological infrastructure and other barriers due to the virtual consultation challenge the successful implementation of telehealth in India, it has the potential to bridge the rural-urban healthcare divide with cost-effective and easily accessible services.

**Conclusion:**

High patient/provider satisfaction underscores the need to integrate telehealth into routine healthcare practices in the country. However, the review urges the government and healthcare practitioners to address the telehealth challenges with prime importance to ensure quality healthcare throughout the nation even after the pandemic.

**Supplementary Information:**

The online version contains supplementary material available at 10.1186/s12913-022-08970-8.

## Background

Respiratory viral pandemics are a massive challenge for nations and their healthcare systems. Research on earlier pandemics revealed that various approaches, such as non-pharmaceutical interventions, healthcare policies, mental health interventions, communication strategies, epidemiological surveillance strategies, and workplace and university measures employed by different countries could limit the spread of viral infections [1]. Similarly, the recent COVID-19 pandemic has also posed unanticipated hurdles to public healthcare systems across the world [2]. Based on previous experience, many countries enacted a variety of pharmaceutical (e.g., vaccination) and non-pharmaceutical (e.g., curfew) interventions as COVID-19 containment measures [3]. Yet, most of their available healthcare resources had to head off to deal with virus-related emergencies [4]. Additionally, the smooth functioning of the healthcare system was hampered due to patients’ and healthcare workers’ fear of infection and mobility restrictions [5]. Apparently, social isolation and lockdown made medical assistance difficult and people’s lives miserable [6]. Unfortunately, individuals with various illnesses, such as hypertension, diabetes, joint and musculoskeletal ailments, and neurological diseases, had limited access to healthcare services during the lockdown [7]. However, lockdowns got extended as most COVID-19 infections are caused by asymptomatic carriers [8]. As a result, reducing in-person interactions with patients became indispensable. Thus, there has been an urgent need to discover new ways to provide healthcare. This necessity has resulted in the rapid implementation and expansion of telehealth services worldwide [9, 10], and healthcare providers have begun to switch from traditional in-person consultations to telehealth [11, 12].

Telehealth refers to the use of digital communication technologies and telecommunications to provide and facilitate healthcare services such as provider and patient education, medical care, health-related information services, and self-care [13]. Some distinguish telehealth from telemedicine, with the former referring to all services provided by health professionals, such as nurses and pharmacists, and the latter restricted to physician-provided health services [14]. However, for this study, telemedicine is used as a subset of telehealth, and the review mainly focuses on delivering healthcare services via information and communication technologies (ICT) during the COVID-19 pandemic. Literature on earlier respiratory viruses suggested the use of telephone-based facilities and media to cope with the pressures of healthcare during pandemics [1]. According to a recent review, telehealth is a useful and popular technology for COVID-19 patients because it is portable, simple, and small [15]. Further, another review demonstrated teleconsultations as an application of internet of things (IoT) and could be used for behavioural and therapeutic adjustments, improving the quality of life of patients, training them about COVID-19, and follow-up [16]. Telehealth is especially beneficial in non-emergency/routine medical care and when direct patient-provider contact is not required, such as delivering psychological services [17]. Telehealth technology is found to be patient-centred, safeguards patients, doctors, and others [18, 19], and provides complete access to caregivers [20]. As a result, telehealth is appealing, affordable, and effective healthcare technology [21–23].

With the substantial urban-rural gap, India went through an unimaginable healthcare crisis during the COVID-19 pandemic. While cities employ 75% of qualified consulting doctors, only 23% work in semi-urban areas and 2% in rural areas [24]. Furthermore, the most disadvantaged and marginalised were publicly supported in primary and secondary healthcare [25]. However, the beneficiaries had limited access to these health facilities due to the COVID-19 restrictions. Thus, the pandemic has underscored the paradigm shift in the country’s healthcare system by switching to telehealth. However, providing the proper directives for telehealth services is crucial as its effective utilisation can substantially change how the country’s healthcare system works. Therefore, it is a dire need to understand the application of telehealth even after the pandemic. Hence, exploring telehealth’s role among medical professionals and patients is of utmost importance [26].

Although there are systematic reviews published on the diverse utilisations of telehealth during the COVID-19 pandemic [27, 28], no such studies specifically focused on the Indian context. On the other hand, India was among the first nations to quickly adopt telehealth to provide healthcare in response to the widespread disruptions due to the COVID-19 pandemic. Nevertheless, a recent study from the country used a scoping review methodology to identify the challenges in providing primary care via telemedicine technology [29]. However, a detailed investigation of telehealth utilisation in multidisciplinary primary and secondary healthcare has received minimal attention. Furthermore, a systematic literature review presents extensive evidence on the contribution of telehealth to the detection, management, and control of illnesses during and after the COVID-19 pandemic remains lacking. Therefore, the present study aimed to understand the role of telehealth-assisted healthcare delivery in the context of the COVID-19 pandemic in India and its applicability even after the pandemic. This review could systematically synthesise and integrate information related to the operation of telehealth services in the country from all available sources. Further, the findings could underscore the need to formulate policies to bolster telehealth services, initiate government-level telehealth services, train healthcare professionals and patients, coordinate awareness programmes, and expand smartphone use or network access among the Indian rural population.

The review question was: what are the applications, benefits and challenges of telehealth in India during the COVID-19 pandemic and beyond?

## Methods

This systematic review was structured adhering to the updated guidelines for reporting systematic reviews (S1 Checklist) [30]. A systematic review methodology was chosen to provide a rigorous and replicable approach to critically analysing the available literature.

### Eligibility criteria

The following inclusion criteria were used. Studies were included if they (a) focused on telehealth users or providers in India, (b) investigated the role of telehealth in any medical speciality during the COVID-19 pandemic, and (c) were published in English. Notably, taking telehealth as a broad concept, studies were selected if they used any form of information and communication technology (ICT) based healthcare services to diagnose, control or manage any diseases/illnesses. However, conference abstracts, secondary data, commentaries, editorials, brief reports, short communication and articles with insufficient data were excluded from the final analysis. Since the present review focused on the COVID-19 pandemic, the year of publication was limited between 2019 and 2021.

### Search strategy

A systematic search was carried out on electronic databases PubMed, Science Direct, Scopus, and Web of Science in February 2022. This comprehensive search was complimented by a manual search on Google Scholar. Several synonyms were used to capture as many studies published on telehealth. Boolean operators “AND” and “OR” were applied to combine the keywords. For instance, the search strategy used in PubMed was (“telehealth” [Title/Abstract] OR “telemedicine” [Title/Abstract] OR “teleconsultation” [Title/Abstract] OR “telecare” [Title/Abstract] AND “covid” [Title/Abstract] OR “covid-19” [Title/Abstract] OR “novel coronavirus” [Title/Abstract]); in Scopus TITLE-ABS-KEY (“telehealth”) OR TITLE-ABS-KEY (“telemedicine”) OR TITLE-ABS-KEY (“teleconsultation”) OR TITLE-ABS-KEY (“telecare”) AND TITLE-ABS-KEY (“covid”) OR TITLE-ABS-KEY (“covid-19”) OR TITLE-ABS-KEY (“novel coronavirus”); and in Web of Science ((TI = “telehealth*”) OR TI = “telemedicine*”) OR (TI = “teleconsultation*”) OR (TI = “telecare*”)) AND ((TI = “covid*”) OR (TI = “covid-19*”) OR (TI = “novel coronavirus*”)).

### Selection process and data extraction

The first author (RE) has done the study conceptualisation and identified the keywords. The first and second authors (RE & AG) simultaneously did an independent search for relevant articles in databases. Next, three authors (ANM, GSR & PK) screened the articles based on study titles and abstracts. After the initial screening, the first four authors independently assessed the remaining studies for full-text analysis and did the data extraction. The last four authors assisted in drafting the manuscript. Significant information was extracted from each finalised article: authors' details, where the study was conducted, study objectives, study design, sample information, and the major findings regarding telehealth applications, benefits, and challenges. Any disagreements during the study selection and review were settled by discussion.

### Quality assessment and evidence synthesis

The methodological quality of included reports was assessed with the JBI appraisal tools [31]. Several study domains were scored on a rating scale of ‘yes’, ‘no’, ‘unclear’ and ‘not applicable’. The first two authors (RE and AG) appraised the studies, and a third reviewer (AJG) decided on discrepancies. A narrative synthesis of extracted information compared and contrasted the ample evidence and qualitatively presented as themes. The reviewers frequently met to discuss the study findings and reached a consensus.

## Results

### Study selection

**Fig. 1:**
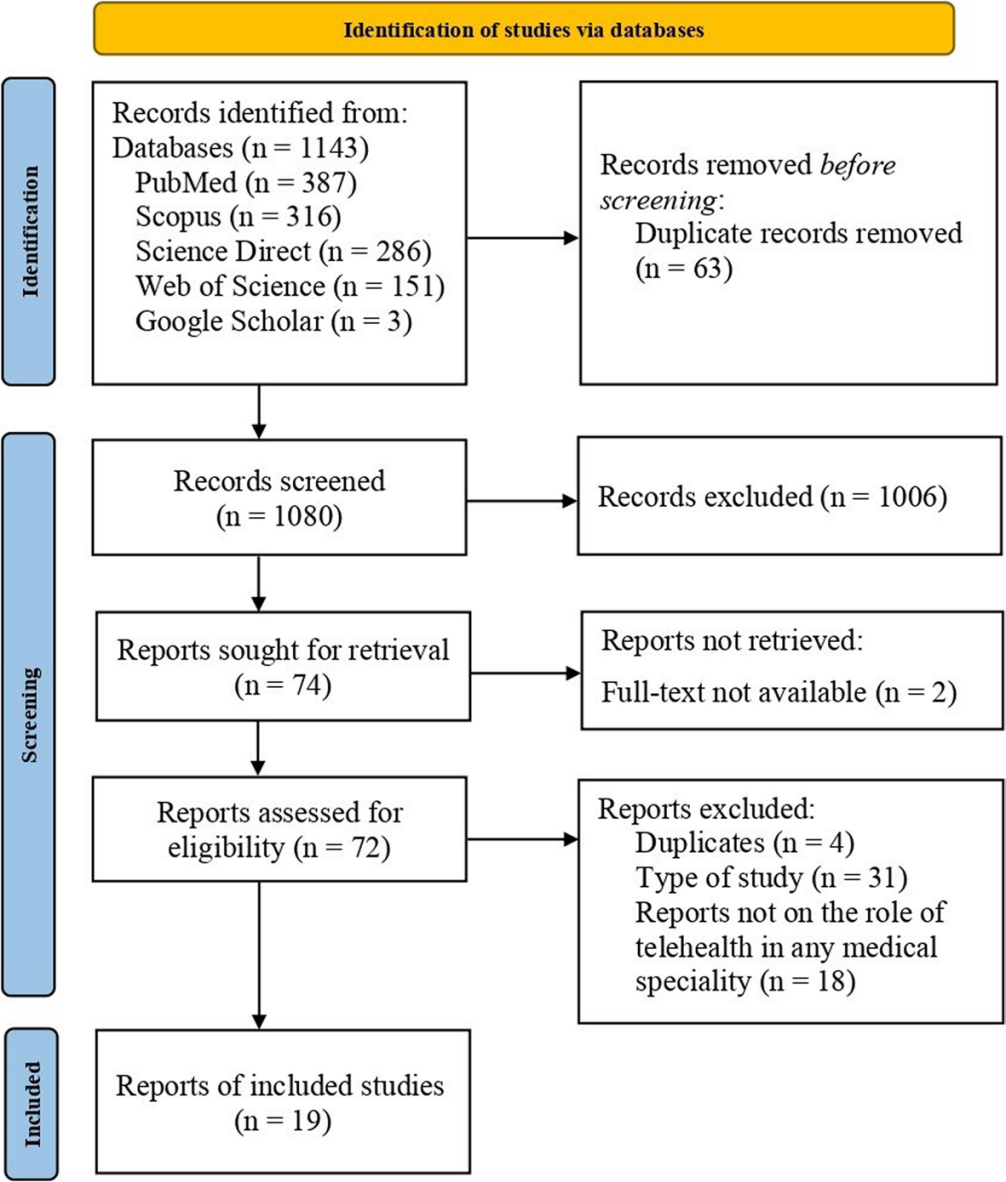
The PRISMA flow diagram

A total of 1143 articles were yielded in the primary databases search. Of these, 387 were from PubMed, 316 from Scopus, 286 from Science Direct, 151 from Web of Science and three from Google Scholar. The entire search process for the systematic review is illustrated in the PRISMA flow diagram (Fig. 1). After removing 63 duplicates, the remaining 1080 records were screened based on eligibility criteria. Subsequently, 1006 studies were eliminated after the title and abstract screening. Out of 74 reports sought for retrieval, two were not retrieved because full text was unavailable. The remaining 72 reports were assessed for eligibility, and 53 studies were removed because they did not meet the inclusion criteria. Thus, the final synthesis included 19 studies emphasising the role of telehealth services across various medical specialities in Indian contexts during the COVID-19 pandemic.

### Study characteristics

The final sample for the present review included 19 studies (Table 1). Among these finalised articles, ten were published in 2020 [32–41], and nine were published in 2021 [42–50]. Included studies consisted of eleven cross-sectional studies [32, 33, 35–37, 39, 40, 45, 46, 49, 50] and eight cohort studies [34, 38, 41–44, 47, 48]. Studies covered the application of telehealth services for different medical issues, including ophthalmology (*n* = 4) [32, 39, 41, 46], neurology (*n* = 4) [38, 42, 45, 50], dermatology (*n* = 2) [35, 43], non-communicable diseases (*n* = 4) [33, 44, 48, 49], haematological illness (*n* = 1) [37], orthopaedic issues (*n* = 2) [36, 47], respiratory illness (*n* = 1) [34], and substance use disorders (SUDs) (*n* = 1) [40]. The current study findings offer a comprehensive understanding of telehealth services during the COVID-19 pandemic in India. Results were further synthesised into three main themes: *applications, benefits and challenges of telehealth services.*

**Table 1:** Summary of study characteristics

Sl. No.	Author(s) & Year	Location of study	Objectives	Type of study	Study population	Sample size	Applications	Benefits	Challenges
1	Das et al., 2020 [32]	India	Examine the teleconsultation experience of patients.	Cross-sectional study	Ophthalmology patients	*n* = 2805	Timely response to eye problems through the use of electronic medical records (EMR).	Positive feedback from the majority of patients.	Lack of EMR system.
2	Anjana et al., 2020 [33]	India	Evaluate the lockdown effects on the adoption of newer technologies.	Cross-sectional study	Type 2 diabetes patients	*n* = 2510	Provided diabetes education. Easy accessibility to diabetes care.	Patient satisfaction. Access to specialists. Remote care. Reduce travel time and cost.	Lack of physical touch. Lack of clarity in the legal framework.
3	Kumari et al., 2020 [34]	North India	Assess the use of telemedicine in follow-up care.	Cohort study	Children with respiratory illnesses	*n* = 188	Successful follow-up care for children who have respiratory problems.	Recommend to others. Time and cost-saving.	Difficulty in phone communication. Connectivity issues.
4	Bhargava & Sarkar, 2020 [35]	India	Evaluate the usage, opinions and attitudes of dermatologists on virtual consultations.	Cross-sectional study	Dermatologists	*n* = 260	New and follow-up patients. Useful for the vulnerable and older population	High physician satisfaction. Easy accessibility. Cost-effectiveness	Difficult to understand patient needs. Internet speed. Technological illiteracy.
5	S. Kumar et al., 2020 [36]	India	Evaluate the telemedicine effectiveness in follow-up care and patient satisfaction.	Cross-sectional study	Orthopaedic patients	*n* = 450	Successful follow-up of orthopaedic patients.	High patient satisfaction Reduces long-distance travel and related costs and discomfort	Lack of physical examination. Failed patient-provider communication. Network issues Unfamiliarity with video calls. Suitability of devices
6	P. Kumar et al., 2020 [37]	North India	Evaluate telemedicine feasibility and different factors that contribute to effective teleconsultation.	Cross-sectional study	Patients with different haematological illnesses	*n* = 944	Follow-up care. Provided advice on drug dose modification or continuation of therapy.	Avoid long travel and tiring journey. Save man-hours. Decrease school/office holidays. Reduce overcrowding.	Challenges in drug availability. Lack of physical examination. Lack of smartphones and internet facility. Limited patient education. Proper surroundings for communication.
7	Panda et al., 2020 [38]	Uttarakhand (North India)	Investigates the efficacy and feasibility of advanced techniques of telecommunication.	Cohort study	Children with various neurological disorders	*n* = 153	Clarified queries of caregivers including drug availability and dosage and commercial brands.	A feasible and effective option for providing medical advice.	Lack of smartphones. Slow-speed internet. Inadequate technical knowledge. Fake practitioners.
8	Pandey et al., 2020 [39]	North India	Examine the feasibility, patient’s clinical profile and addressability using teleconsultations.	Cross-sectional study	Ophthalmologists	*n* = 32	About 60% of consultations were successfully managed without physical examination.	Virtual advice is sufficient for many eye care problems.	Legal concerns. Unavailability of technology. Lack of trust in modality. Data confidentiality. Poor means of communication. Unawareness among rural people.
9	Sahu et al., 2020 [40]	India	Evaluate the acceptability of e-consult for substance use disorder management.	Cross-sectional study	Health care providers (HCPs)	*n* = 68	Receive guidance from specialists.	Increased accessibility. Time-saving. High HCP satisfaction.	Patient privacy. Not as suitable as in-person consultations.
10	Agrawal and Agarwal, 2020 [41]	Indore, Madhya Pradesh	Analyse the impact of teleophthalmology in the management of eye diseases.	Cohort study	Patients with eye problems	*n* = 119	Diagnosis and management of most of the cases of tele ocular surface disorders.	Advancement of digital media. Easy availability of smartphones.	Not effective in treating eye diseases that need intervention.
11	Garg et al., 2021 [42]	Delhi	Assess the feasibility of tele neurorehabilitation (TNR) in low-resource contexts.	Cohort study	Patients with Parkinson’s disease	*n* = 22	The TNR intervention was shown to be safe, with no serious consequences.	TNR is a feasible option among patients with Parkinson’s disease	Shared smartphones. Wi-Fi bandwidth issues. Lack of rapport. Inadequate technical skills. Poor hand-motor skills.
12	Handa et al., 2021 [43]	North India	Analyse patient-physician experience and acceptance of teledermatology	Cohort study	Dermatology patients Dermatologists	*n* = 6125 *n* = 34	The diagnosis was ascertained online in 93.45% of cases. 62% of acne patients reported being satisfied or very satisfied.	High satisfaction among new and follow-up patients	Duplicate entries. Privacy concerns Connectivity issues. Patient technological inability. Lack of rapport.
13	Mishra et al., 2021 [44]	Haryana	Examine the feasibility of telemedicine-based diabetes education	Cohort study	Hospitalised diabetes patients with COVID-19	*n* = 100	Telemedicine was accepted and appreciated by 96.0% of patients.	Effective means to provide diabetes education	Lack of internet services. Difficulty following medical advice via telephone. Difficulty operating smartphones.
14	Nair et al., 2021 [45]	South India	Assess the satisfaction, feasibility and effectiveness of teleconsultation	Cross-sectional study	Persons with epilepsy	*n* = 141	Successful video consultations with no additional cost. Prescribed new drugs.	An acceptable and effective method to follow up on persons with epilepsy. Effectively conveyed medical advice.	Poor connectivity in rural areas. Lack of smartphones. Security and privacy concerns.
15	Ravindran et al., 2021 [46]	South India	Describe the teleconsultation experiences of patients during COVID-19 lockdown	Cross-sectional study	Ophthalmology patients	*n* = 621	Treatment and management of eye care diseases for both new and follow-up patients.	Ensure continuity of care during the lockdown. Save travel time. Avoid overcrowding.	Poor network connectivity. Medicolegal implications. Data privacy and confidentiality.
16	Sandhu et al., 2021 [47]	North India	Discuss the telemedicine services available for patients during the COVID-19 pandemic.	Cohort study	Rheumatoid arthritis patients	*n* = 74	New and follow-up care. Medical advice regarding prescription changes and related issues.	Use in future. Recommend to others. Saves waiting time at hospitals. Eliminates travel time. High patient satisfaction.	Language of clinicians. Unfamiliar technology. No personal phone. Poor network connectivity. Lack of clarity of medical advice. Lack of expertise.
17	Adhikari et al., 2021 [48]	India	Evaluate the feasibility of telemedicine-based palliative interventions.	Cohort study	Advanced stage Cancer Patients	*n* = 547	Follow-up care. Evaluation of treatment response. Prognostication.	High patient satisfaction. Chronic pain assessment and symptomatic supportive care.	Voice/video quality. Advice clarity. Language.
18	Ullas et al., 2021 [49]	South India	Investigate the adoption rates and perception of telemedicine.	Cross-sectional study	Patients with non-communicable diseases.	*n* = 220	New and routine follow-up care for non-communicable diseases	Comfortable to use. An adequate surrogate for in-person consultations.	Difficult to get an appointment with a regular doctor or obtain medicine on time. Data privacy risk. Technological unfamiliarity. Limited connectivity.
19	Raheja et al., 2021 [50]	India	Evaluate the current telemedicine practice gaps and address them for future consultations.	Cross-sectional study	Neurosurgical patients who used telemedicine facility	*n* = 231	97% of patients reported telemedicine in neurosurgery as beneficial.	Less travel expenditure. Reduced time and resources.	Poor network. Lack of physical examination. Reduced communication/discussion. Misinterpretation of prescription.

### Quality assessment

The quality appraisal of 19 included studies was done using JBI critical checklists. The risk of bias for assessed reports was generally moderate to high, mainly due to the lack of valid outcome measures. Also, no studies were removed based on the level of methodological quality. The results of the quality assessment can be found in the S2 File.

### Applications of telehealth across medical specialities

The findings from the included studies highlight the application of telehealth services in diagnosing and managing different medical complaints related to ophthalmology, neurology, dermatology, non-communicable diseases, haematological illness, orthopaedic issues, respiratory illness and substance use disorders (SUDs).

#### Ophthalmology

The telehealth application in assisting patients with eye problems was presented in four studies [32, 39, 41, 46]. Notably, electronic medical records allowed for a quick response to patients with ophthalmology issues during the COVID-19 pandemic lockdown [32]. More than 80% of the consultations were from patients who had previously visited the clinic, and about 60% of ophthalmology patients were not prescribed for physical evaluation in person [39]. Teleophthalmology was used for both diagnosis and management of eye problems, especially any damage to the surface layers of the eye, namely the cornea and conjunctiva [41]. Further, it ensured continuity of care during the pandemic as a means of managing new as well as follow-up patients with most eye conditions [46].

#### Neurology

Synchronous video consultations using mobile phones were reported as an effective mode of telehealth delivery for follow-up patients with epilepsy [45]. Raheja et al. found that 82% of neurosurgery patients reported that teleconsultation call was made on time as per the allotted appointment schedule, and the majority of patients (97%) felt that it has at least one advantage [50]. While considering virtual interventions, teleneurorehabilitation was a viable choice for people with parkinson’s disease during the current pandemic as it was shown to be safe, with no serious consequences [42].

#### Dermatology

The application of telehealth in dermatology was more practical and easier to adopt as it is largely a visual area, and most disorders can be detected with a simple inspection and a few leading questions [35]. They also reported that teledermatology could be utilised for new and follow-up patients as well as vulnerable and older populations. Handa et al. revealed that diagnosis was made in 93.45% of cases through teleconsultations, and only 2.2% of cases were recommended for in-person visits, especially none of the patients with ectoparasitic, dermatophyte or acne infections required face-to-face consultation [43].

#### Non-communicable diseases

Patients with non-communicable diseases were more amenable to teleconsultations during the COVID-19 pandemic because of the shortened time for consultations, the difficulty in scheduling in-person visits, and the lessened physical evaluation during consultations [49]. Another study suggested that telemedicine-based diabetes education is an effective, viable and acceptable technology in the COVID-19 management of most patients with type 2 diabetes [44]. However, Anjana et al. concluded that the acceptance of telemedicine facilities is still suboptimal despite their importance in delivering diabetes education and the high levels of satisfaction among the users [33]. In their study, Adhikari et al. reported that teleconsultation could resolve most complaints of advanced cancer patients with a single-time call [48].

#### Haematological illness

One study considered the application of telehealth services to manage patients with haematological diseases [37]. They reported that three-fourths of patients used telemedicine for regular follow-up advice regarding the continuation of therapy or drug dose modifications. Successful consultations included patients with chronic myeloid leukaemia acquired or inherited marrow failures, myeloma or lymphoma and anaemia.

#### Orthopaedic conditions

Findings from a study reveal that telecall-based consultations in orthopaedics can be successfully applied to maintain follow-up care, particularly for patients with back pain and cervical complaints, which account for more than one-third of follow-up patients [36]. In another study, Sandhu et al. found telemedicine a feasible method to manage rheumatic diseases and highlighted a greater acceptance of telemedicine even though many patients had never seen or communicated over the internet [47].

#### Respiratory illness

One study assessed the role of teleconsultation in the follow-up care of children with respiratory problems [34]. The study suggested that telemedicine can successfully manage most children with respiratory complaints who receive follow-up care. They did not encounter any issues during teleconsultations, and 80% of the caretakers reported high satisfaction with telemedicine services.

#### Substance use disorders (SUDs)

In their study, Sahu et al. employed an asynchronous e-consult-based teleconsultation for substance use disorders, which connects healthcare providers (HCPs) with addiction specialists, found easy access of the majority of HCPs to the specialist, increased clinical care, and high clinician satisfaction as they are not required to meet real-time [40].

### Benefits of telehealth in India

The majority of included studies highlighted the benefits of telehealth and stated that it would stay in the country’s healthcare system even after the pandemic [37, 47]. The main reasons for continuing teleconsultation were grouped into five subthemes: patient-provider satisfaction, cost-effectiveness and easy accessibility, new and follow-up care, instant medical advice, and bridging the urban-rural healthcare divide.

#### Patient-provider satisfaction

Included studies reported high satisfaction of patients [33, 36, 43, 47, 48], as well as the providers [35, 40] with telehealth services. Das el. in their study found that 82% of patients who used teleconsultations were happy with their experience, and 58.1% were willing to continue the use in the future [32]. More than half of the respondents in a study reported a future for teleconsultations in their routine practice of dermatology care as palpation and detailed physical examination are less required to diagnose and treat dermatological problems [35]. Further, findings revealed that patients/providers would recommend telehealth to others [34, 47].

#### Cost-effectiveness and easy accessibility

Teleconsultations provide access to quality healthcare at the patient’s doorstep [33]. Findings revealed that those who have preferred to continue telehealth services stopped in-person consultations during the pandemic to avoid exposure risk and thus saved time and money on hospital visits [33, 34, 47, 50]. It reduced the inconvenience caused due to long travel distances, expenses, and further discomfort to the patients [36, 42]. In another study, healthcare providers reported easy access to specialist care through e-consult and viewed it as an excellent addition to regular consultation [40].

#### New and follow-up care

Teleconsultations in the country were found to have benefits like more convenient follow-up [34, 37, 43, 45–47], thereby reducing overcrowding in hospitals [34, 37]. One study on teledermatology reported that earlier teleconsultations focused mainly on follow-up care, while virtual care was provided for new and follow-up patients during the pandemic [35]. Telehealth-based healthcare services were delivered successfully for new and follow-up patients [46, 49]. During follow-up calls, most patients asked for advice regarding prescription change and related complications, while teleconsultations of new patients included screening for disease signs, documentation and video conference if required [47].

#### Instant medical advice

Caretakers of children with epilepsy largely used teleconsultations to obtain medical advice, and their queries were mainly related to anti-epileptic drug usage and modification, commercial brands, routine visits, and concerns about the adverse effects of COVID-19 on epileptic children [38]. In a study, 74.3% of regular follow-up patients needed advice about drug dosage modification or continuation of treatment [37]. The most common advice given to the patients in another study was related to medications, followed by appointment-related queries and fixing surgical appointments [40]. Relatedly, in their study, Nair et al. successfully contacted post-epilepsy surgery cases and could provide advice about the tapering of drugs [45]. Whereas Adhikari et al. reported that changes in drug dosage or formulation or the prescription of a new medication were included in the most common medical advice offered to patients [48].

#### Bridging urban-rural healthcare divide

Lockdown has impaired the already difficult transportation of patients in geographically challenging regions [38]. Results from a survey of dermatologists revealed that almost 82% of respondents practised in an urban setting, and 50% had a private clinic of their own [35]. At the same time, teleconsultations have the potential to be accessed by patients in remote areas [32, 34–36, 41, 50]. Virtual consultations save money and effort, particularly for patients from the rural areas as they do not have to travel long distances to get consultation and treatment [35]. Most rural and semi-urban parts of the country have access to the internet and telecommunications facilities. As a result, teleconsultation is becoming more important in reaching out to people with restricted access to healthcare [45].

### Challenges of telehealth services

The included studies identified potential challenges to the successful implementation of telehealth services in the country, which were synthesised into nine subthemes: insufficient medical and technological infrastructure, urban-rural digital divide, technological illiteracy, lack of physical examinations, limited patient-provider relationship, difficulty with appointment and consultation time, lack of experts, data security and privacy, and legal concerns.

#### Insufficient medical and technological infrastructure

Many institutions’ lack of electronic medical records leads to depending on patient-provided reports to the clinician via electronic media such as email or WhatsApp, which impedes the real-time resolution of patient queries [32, 46]. In addition, the unavailability of basic technical infrastructures such as smartphones and the internet is a major barrier to the integration of telehealth into practice [37]. The insufficient technological infrastructure reported in the included studies was lack of smartphones [36, 38, 42, 45] and network connectivity issues [35, 38, 42–45, 50]. One study found that caregivers without well-equipped smartphones could not share clinical reports via picture message or video recording and the medication was prescribed based on the best possible clinical judgment of the consulting doctor [38]. In another study, patients faced problems attending teleconsultations as many shared smartphones with their family members [42].

#### Urban-rural digital divide

Lack of proper internet connectivity in the country’s rural (especially remote hilly) areas may limit healthcare access to socially, economically, or geographically disadvantaged populations of patients in these regions [49]. One study reported that they had infrequent video consultations as most of their patients were from rural areas where internet connectivity was poor [46]. Similarly, another study revealed that some rural areas might experience weak network connectivity [45].

#### Technological illiteracy

Technology illiteracy among the citizens is a potential challenge to implementing a telehealth system in the country [35]. Many of the included studies reported on the technological inability from the patient’s end [43, 47] and unfamiliarity with video calls [36]. In their study, Mishra et al. found that several patients depended on their children to operate video-consultation devices [44]. In another study, patients with poor hand-motor skills faced difficulty using a smartphone, while none felt technologically skilled enough to handle a computer to manage this concern [42].

#### Lack of physical examinations

One of the challenging factors for telehealth is the lack of physical examination [37, 47, 50]. One study revealed that while a lack of physical examination could be justifiable for new patients, it may not always be suitable for patients who need follow-up care as a superficial knowledge of the general condition and the symptom progression can guide further treatment [36].

#### Limited patient-provider relationship

Telehealth lacks the human touch, one of the main prerequisites of an effective doctor-patient relationship [33]. Additionally, teleconsultations face reduced opportunities to show empathy to patients, understand their needs, and adequately counsel them [35]. In their study, Garg et al. reported that patients expressed inadequate rapport or sense of belonging that they otherwise feel after visiting the doctor in person [42]. Similarly, another study found suboptimal verbal communication and discussion as a limitation of telehealth services [50].

#### Difficulty with appointment and consultation time

Phone calls at inappropriate timings may contribute to a lack of interest in teleconsultations and a contributing reason some patients decline such services in the first place [36]. Whereas another study reported that treatment got affected as patients could not get an appointment with their routine doctor, possibly leading to patient dissatisfaction [49]. Moreover, about one-fourth of the patients in a study felt that pre-set time limit for teleconsultations restricted documenting their medical history in detail [36].

#### Lack of experts

In their study, Panda et al. reported that there are now only a few trained radiologists and radiologists in India who are well-versed in magnetic resonance imaging (MRI) and electroencephalogram (EEG) brain reporting, respectively, primarily locate in urban areas [38]. Further, they extend that while MRI images can be telemetrically transferred over a long distance, tele-EEG transportation is still out of reach for most places. Whereas in another study, 61.76% of healthcare providers reported low acceptability of e-consultations with medical specialists as they were concerned with the specialist’s expertise level [40]. The study further stated that addiction specialists in developing countries like India are very few, predominantly located in public sector teaching hospitals and busy with clinical services.

#### Data security and privacy

Although technological infrastructure is available, data security and privacy are major challenges to telehealth [36, 45]. Patient-provider apprehension about data confidentiality needs to be worked through with prime importance [39, 49]. There is no option to know if the mobile health application developer has taken the necessary precautions to ensure the privacy and security of the application against malicious attacks [45]. Adhikari et al. reported that the major limitation of using these mobile-based applications is the data safety of the patients [48].

#### Legal concerns

Pandey et al. revealed that 15.7% of doctors were reluctant to use teleophthalmology due to their concerns regarding the associated legal liabilities [39]. There is a potential risk of diagnostic errors and subsequent medicolegal implications [39, 46].

## Discussion

This systematic review aimed to synthesise current evidence on the role of telehealth services in India during the COVID-19 pandemic. Results reveal that the virus outbreak and the resulting lockdown have significantly impacted the country’s healthcare system [39]. Surprisingly, the shift to telehealth was well accepted by healthcare professionals and patients. The results demonstrate a broad application of telehealth services to various medical specialities. These findings are in line with a review that revealed the utilisation of telehealth during the current pandemic to control and assess multiple diseases such as diabetes, epilepsy and cancer [27].

Telehealth is depicted in the literature as a feasible tool for delivering health services with high patient-provider satisfaction. The benefits include cost-effectiveness and easy accessibility, new and follow-up care, and instant medical advice. These findings support existing literature on teleconsultation satisfaction for convenience, shorter waiting times, avoidance of unnecessary travelling times and costs, and time off work [51]. Furthermore, studies within this review revealed cost savings that were particularly magnified if patients had to travel long distances. Only 37% of India’s rural population within 5 km can access inpatient services, while 8% of patients can access outpatient healthcare facilities [52]. Findings highlight the role of telehealth in bridging the rural-urban healthcare divide by delivering healthcare services in resource-constrained settings of the country. Consistent with these findings, existing literature claims that the aim to reduce healthcare costs, particularly for patients who reside distant from their healthcare facility, was a driving force for the implementation of telehealth [53–55].

However, evidence suggests that insufficient medical and technological infrastructure and technical illiteracy are potential challenges to telehealth. Similar results were found in other studies that reported technical and technological issues were major barriers to the implementation and adoption of telehealth [27, 56–58]. Furthermore, many patients had to share smartphones with their family members, which supports related research that some patients lack appropriate gadgets in their homes to access telehealth [58]. Additionally, findings reveal that virtual consultation leads to challenges such as lack of physical examination, limited patient-provider relationship and difficulty with appointment and consultation time. Corroborating these findings, other studies also reveal that physical examination can be accomplished only through face-to-face consultation and not by telehealth [59–62]. Evidence also suggests that a lack of experts and necessary equipment may hinder successful telehealth implementation. In contrast, the installation of expensive equipment can be justified as it holds the potential to minimise healthcare costs for patients in rural areas [63]. Also, patient/provider apprehension regarding data security, privacy, and other legal concerns challenge telehealth services. The safety risks of telehealth applications and the usage of telecommunication directly impact patient data privacy [58]. Inadequate medicolegal considerations to assist telehealth was reported as a critical barrier to the adoption of telehealth across different countries [64].

This systematic review presents comprehensive evidence on the role of telehealth technology in India within the COVID-19 pandemic and in the future. Although available reviews report the challenges in delivering primary care using telemedicine in the country during the pandemic [29], the current study demonstrates the applications, benefits and challenges of telehealth, including telemedicine across various medical specialities in primary and follow-up care. Further, the study proposes some crucial recommendations for effectively implementing telehealth practices in the country. Given the potential applications and benefits of telehealth services, the government and policymakers should try to improve technological infrastructure across the country as well as the technological literacy of the population in this era of digitalisation. Data privacy and security should be strictly maintained as these could influence the decision of patients, caregivers and healthcare providers to adopt telehealth services. Further, improved documentation and adherence to information management principles could help healthcare providers avoid medicolegal issues. All reasonable efforts should be made to deliver high-quality patient-centred care to benefit the nation from telehealth services irrespective of social, economic and geographical differences.

The present review included cross-sectional and cohort studies. Nevertheless, results were supported equally by both forms of evidence, and the inclusion of these reports enabled a comprehensive presentation of findings. However, the studies varied in their methodological quality. Notably, several studies omitted participants due to their lack of access to telehealth technology and poor language skills, leaving out a group of patients who perhaps were less satisfied with telehealth. Similarly, the included studies can have a selection bias because patients who consented to join telehealth studies were more likely to be aware of technical operations. Additionally, many studies examining patient/provider satisfaction used non-validated measures, which compromised the validity of the findings. Other studies deemed low quality by JBI appraisal checklists neglected to account for confounding factors that can influence the patients’ condition during the study period. Given the relevance of telehealth in the country, future studies can focus on developing a validated measure to assess provider and patient satisfaction for reliable and comparable study findings. Moreover, confounding factors such as extended time periods between teleconsultations can be explored further.

Furthermore, there are some limitations to this systematic review. Even though several technologies are being deployed for telehealth, the current review attempted to include all studies focused on the role of telehealth across various medical specialities during the COVID-19 pandemic in India. Nevertheless, these studies could cover all components and activities of healthcare and the healthcare system conducted through telecommunication technologies. In addition, the heterogeneity of patient/provider populations in the review sample makes the direct comparison of findings difficult. Therefore, it is difficult to determine the actual scope of telehealth. However, focusing on various medical specialities offers current insight into the wide-ranging application of telehealth within the Indian healthcare system. Although the present review employed a comprehensive search strategy and attempted to cover a wide range of evidence on telehealth services in the country, some articles on these themes in the literature could miss the analysis.

## Conclusion

The current systematic review provides comprehensive evidence on the role of telehealth in ensuring continuity of care during the COVID-19 pandemic in India. The findings support the practical application of telehealth services for diagnosing and managing various medical complaints, including ophthalmology, neurology, dermatology, non-communicable diseases, haematological illness, orthopaedic issues, respiratory illness and substance use disorders (SUDs). The overall positive attitude and satisfaction of patients, caregivers and healthcare professionals with telehealth services due to its easy accessibility, time and money-saving expand the scope that lies ahead even after the pandemic. However, the technological challenges and other potential barriers that can impede the growth of telehealth should be addressed within the medical community to make it a viable option for the present and future healthcare practice in the country. Moreover, future research can explore telehealth’s advantages, validity and effectiveness in each medical speciality, mainly during an era of limited physical patient contact and even after that. Further, studies can also evaluate the facilitators and barriers to patient-provider satisfaction with telehealth services.

## Supplementary Information

**Additional file 1.**

**Additional file 2.**

## Data Availability

All data relevant to the study are included in the article or uploaded as supplementary information.
