# Supplementary material for: Applications, benefits and challenges of telehealth in India during COVID-19 pandemic and beyond: a systematic review
Source: BMC Health Serv Res. 2023 Jan 4;23:7. doi: 10.1186/s12913-022-08970-8 (PMC9810518; doi:10.1186/s12913-022-08970-8)
Supplement: Supplementary file 2 — Additional file 2. [file 12913_2022_8970_MOESM2_ESM.docx]

**Supplement 1 Quality assessment results of cross-sectional studies using Joanna Briggs Institute (JBI) Checklist**

| **Author(s) & Year** | **Q1^a^** | **Q2** | **Q3** | **Q4** | **Q5** | **Q6** | **Q7** | **Q8** | **% Yes** | **Risk^b^** |
| --- | --- | --- | --- | --- | --- | --- | --- | --- | --- | --- |
| Das et al. (2020) [25] | Y | Y | N | N | N | N | N | Y | 37.5 | High |
| Anjana et al. (2020) [26] | Y | Y | N | Y | N | N | Y | Y | 62.5 | Moderate |
| Bhargava and Sarkar (2020) [28] | Y | Y | N | N | N | N | N | Y | 37.5 | High |
| S. Kumar et al. (2020) [29] | Y | Y | Y | Y | N | N | Y | Y | 75 | Low |
| P. Kumar et al. (2020) [30] | Y | Y | Y | Y | U | N | N | Y | 62.5 | Moderate |
| Pandey et al. (2020) [32] | Y | Y | N | U | N | N | N | Y | 37.5 | High |
| Sahu et al. (2020) [33] | Y | Y | U | U | N | N | Y | Y | 50 | Moderate |
| Nair et al. (2021) [38] | Y | Y | Y | Y | N | N | U | Y | 62.5 | Moderate |
| Ravindran et al. (2020) [39] | Y | Y | Y | Y | N | N | U | Y | 62.5 | Moderate |
| Ullas et al. (2021) [42] | Y | Y | Y | Y | Y | U | Y | Y | 87.5 | Low |
| Raheja et al. (2021) [43] | Y | Y | Y | Y | N | N | Y | Y | 75 | Low |

^a^Q1 – Q8 indicates questions 1 to 8 as per the JBI appraisal tool.

Y - Yes, N – No, U – Unclear N/A – Not/Applicable

^b^ 49% of “yes” scores were rated as high risk of bias, 50 to 69% of “yes” scores as moderate, and 70% of “yes” scores as low risk of bias.

**Supplement 2 Quality assessment results of cohort studies using Joanna Briggs Institute (JBI) Checklist**

| **Author(s) & Year** | **Q1^a^** | **Q2** | **Q3** | **Q4** | **Q5** | **Q6** | **Q7** | **Q8** | **Q9** | **Q10** | **Q11** | **% Yes** | **Risk^b^** |
| --- | --- | --- | --- | --- | --- | --- | --- | --- | --- | --- | --- | --- | --- |
| Kumari et al. (2020) [27] | U | N | N | N | N | Y | N | Y | U | N | Y | 27 | High |
| Panda et al. (2020) [31] | N | N | N | N | N | Y | N | Y | U | N | Y | 27 | High |
| Agrawal and Agarwal (2020) [34] | N | N | N | N | N | Y | N | Y | U | N | Y | 27 | High |
| Garg et al. (2021) [35] | N | N | Y | N | N | Y | N | Y | U | N | Y | 36.4 | High |
| Handa et al. (2021) [36] | N | N | Y | N | N | Y | N | Y | U | N | Y | 36.4 | High |
| Mishra et al. (2021) [37] | Y | N | Y | N | N | Y | N | Y | U | N | Y | 45.5 | High |
| Sandhu et al. (2021) [40] | N | Y | Y | N | N | Y | Y | Y | U | N | Y | 54.5 | Moderate |
| Adhikari et al. (2021) [41] | N | Y | N | N | N | Y | N | Y | U | N | Y | 36.4 | High |

^a^Q1 – Q11 indicates questions 1 to 11 as per the JBI appraisal tool.

Y - Yes, N – No, U – Unclear N/A – Not/Applicable

^b^ 49% of “yes” scores were rated as high risk of bias, 50 to 69% of “yes” scores as moderate, and 70% of “yes” scores as low risk of bias.
